# SNX9 Orchestrates Lung Metastasis via EGFR-ERK Signaling and Actin Cytoskeleton Remodeling in Breast Cancer

**DOI:** 10.32604/or.2026.082536

**Published:** 2026-09-14

**Authors:** Qingqing Liu, Lei Li, Kumar Ganesan, Yang Jiang, Kewu Zeng, Yue Sui, Xinyuan Guan, Rongfang He, Jianping Chen

**Affiliations:** 1 School of Chinese Medicine, Li Ka Shing Faculty of Medicine, The University of Hong Kong, Hong Kong SAR, China; 2 Shenzhen Institute of Research and Innovation, The University of Hong Kong, Shenzhen, China; 3 Department of Pathology, the First Affiliated Hospital, Hengyang Medical School, University of South China, Hengyang, China; 4 State Key Laboratory of Oncology in South China, Guangdong Provincial Clinical Research Center for Cancer, Sun Yat-Sen University Cancer Center, Guangzhou, China; 5 The Department of Traditional Chinese Medicine, Beijing Jishuitan Hospital, Beijing, China; 6 School of Pharmaceutical Sciences, Peking University, Beijing, China; 7 Department of Clinical Oncology, Li Ka Shing Faculty of Medicine, The University of Hong Kong, Hong Kong SAR, China

**Keywords:** Sorting nexin 9 (SNX9), EGFR-ERK signaling, actin cytoskeleton remodeling, breast cancer (BC)

## Abstract

**Objectives:** Sorting nexin 9 (SNX9) participates in endocytic trafficking and has been connected to several malignancies, but its involvement in breast cancer (BC) remains incompletely resolved. This work was designed to examine whether SNX9 supports BC progression and investigate signaling and cytoskeletal processes associated with its activity. **Methods:** The clinical relevance of SNX9 was assessed using bioinformatics analysis of publicly available cancer databases. Lentiviral vectors were used to establish BC cell models with stable SNX9 overexpression or knockdown. Both cellular (proliferation and motility) and murine (tumor growth and metastatic colonization) experiments were implemented to functionally characterize the SNX9-mediated phenotypes. The underlying mechanisms were investigated via western blotting, immunofluorescence, co-immunoprecipitation, and pathway-focused analyses. **Results:** Across the analyzed datasets, greater SNX9 abundance was linked to worse overall survival outcomes in BC patients. Functionally, SNX9 upregulation conferred increased proliferative, migratory, and invasive capacities *in* *vitro* and contributed to both primary tumor enlargement and distant metastatic spread *in* *vivo*, whereas SNX9 depletion produced the reciprocal phenotypes. SNX9 silencing also increased the G2/M cell fraction and disrupted actin cytoskeletal organization mechanistically linked to reduced Ras-related C3 botulinum toxin substrate 1 (Rac1)/cell division cycle 42 homolog (Cdc42) activation and the subsequent impairment of lamellipodial and filopodial protrusion. Additionally, co-immunoprecipitation substantiated the physical coupling between SNX9 and the scaffold protein tyrosine kinase substrate with five SH3 domains (TKS5), which correlated with the invasive behavior of BC cells. **Conclusion:** Collectively, our findings establish SNX9 as a critical oncoprotein that drives BC progression by coordinating proliferative epidermal growth factor receptor (EGFR)/extracellular signal-regulated kinase 1/2 (ERK1/2) signaling and cytoskeletal dynamics through interactions with TKS5 and Rac1/Cdc42. Clinically, SNX9 qualifies as a promising prognostic classifier and a rational target for therapeutic intervention.

## 1 Introduction

Breast cancer (BC) continues to be a main contributor to cancer fatalities in the female population worldwide, driven by substantial incidence and mortality burdens [1,2,3]. Immunoreactivity of estrogen receptor (ER) and progesterone receptor (PgR), together with human epidermal growth factor receptor 2 (*HER2*) amplification status, defines four clinicopathological subtypes: luminal A (ER+/PgR+, *HER2*−; ~50%), luminal B (ER+/PgR+, *HER2*± with higher proliferation than Luminal A; ~20%), *HER2*-enriched (ER− and PgR−, *HER2*+; ~15%), and triple-negative breast cancer (TNBC; ER−, PgR− and *HER2*−; ~15%) subtypes [4,5]. Despite multimodal treatment advances, including surgical and chemo- or targeted therapies [6], which have improved survival, metastatic dissemination to bone, lung, liver and brain remains the predominant cause of patient mortality [7,8,9]. Consequently, uncovering the functional contributions of specific genes to both primary tumor maintenance and metastatic outgrowth is a critical research priority.

Sorting nexins share a phox homology (PX) domain that selectively binds and recognizes phosphoinositides lipids in cellular membranes [10]. Through their involvement in endosome-lysosome, autophagy-lysosome, and ubiquitin-proteasome systems, SNXs serve as essential guardians of protein homeostasis [11]. Given their pleiotropic regulatory functions, SNX proteins have been associated with an extensive range of pathological conditions, encompassing cardiovascular diseases [12], hypertension [13], neurological disorders [14], and various malignancies [15,16,17]. Among the 33 SNX proteins identified in mammalian genomes, sorting nexin 9 (SNX9) has attracted substantial interest owing to its well-established functions in clathrin-mediated endocytosis and intracellular trafficking and its emerging role as a key modulator of tumor cell phenotypes [18,19]. For example, knockdown of SNX9 inhibited transferrin internalization in HeLa cells [20]. Double knockdown of SNX9 and SNX18 showed remarkably reduced a disintegrin and metalloprotease 9 (ADAM9) internalization [21]. Preliminary findings also found that SNX9 is a new regulator of BC [22,23,24]. For instance, depletion of SNX9 was reported to suppress the endocytosis of integrin β3 to delay adhesion turnover, leading to impeded cell invasion [24], while another study reported that knockdown of SNX9 would also damage the endocytic internalization of MT1-MMP in invadopodia, which helped matrix degradation [23]. Nevertheless, the precise contributions of SNX9 to BC pathogenesis encompassing both its phenotypic effects and the associated molecular networks remain to be fully characterized.

To address this gap, our investigation aims to elucidate the functional relevance and signaling framework of SNX9 in BC progression and metastatic spread by dissecting key downstream signaling pathways, using both *in vitro* functional assays and *in vivo* murine models.

## 2 Materials and Methods

### 2.1 Cell Culture

MCF-7 (HTB-22), BT-549 (HTB-122), MDA-MB-231 (HTB-26), and HEK293T (CRL-3216) were obtained from the American Type Culture Collection (ATCC; Manassas, USA) and authenticated by STR profiling. BT-549 and MDA-MB-231 cells were grown in complete 1× DMEM (Thermo Fisher Scientific, 11995065, Gibco, Waltham, USA) consisting of 10% fetal bovine serum (FBS) (Thermo Fisher Scientific, 10270106) after heat-inactivation and 1% penicillin-streptomycin (P/S) (Thermo Fisher Scientific, 15140122). HEK-293T cultures used the complete DMEM supplemented 1% geneticin (G418 Sulfate) (50 mg/mL) (Thermo Fisher Scientific, 10131035), 1% MEM Non-Essential Amino Acids Solution (100×) (Thermo Fisher Scientific, 11140035), 1% Sodium Pyruvate (100 mM) (Thermo Fisher Scientific, 11360070), and 1% L-Glutamine (200 mM) (Thermo Fisher Scientific, 25030081). MCF-7 cells were cultured in complete RPMI 1640 medium (Thermo Fisher Scientific, 11875093) supplemented with 10% heat-inactivated FBS (Thermo Fisher Scientific, 10270106) and 1% P/S (Thermo Fisher Scientific, 15140122). For daily maintenance, cells were incubated at 37°C in a humidified environment with 5% CO_2_, and periodically tested for mycoplasma contamination.

### 2.2 Plasmids and Lentivirus Transfection

An SNX9 expression construct (NM_016224; pLenti CMV-MCS-EF1A-luciferase-T2A-puro) was obtained from LncBio Co., Ltd. (Shanghai, China). Lentiviral SNX9 short hairpin RNAs (shRNAs) vectors (pLVX-U6-MCS-CMV-luciferase-PGK-Puro) were supplied by Hunan Fenghui Biotechnology Co., Ltd. (Changsha, China); target sequences were listed in Table 1. For lentivirus packaging, HEK293T cells were plated at 8 × 10^4^ cells/cm^2^ and transfected when reaching 70–90% confluency. For each well, solution A was prepared by diluting 6 μL using Lipofectamine™ 2000 (Thermo Fisher Scientific, 11668027) in 150 μL Opti-MEM (Thermo Fisher Scientific, 31985070). Solution B contained a plasmid mixture in 150 μL Opti-MEM, consisting of 0.5 μg pRSV-Rev (Addgene 12253), 0.5 μg pMDLg/pRRE (Addgene 12251), 0.5 μg pMD2.G (Addgene 12259), and 1 μg constructs encoding SNX9 or shRNAs. After separate 5-min incubations, the solutions were combined for 20 min at 22°C and gently applied to the cells in a dropwise manner. Medium was renewed after 4–6 h post-transfection. Lentivirus-containing supernatants collected at 48 h were pooled, cleared at 500× *g* for 5 min, and filtered through a 0.45-μm pre-sized PES membrane. Target cancer cells (1–2 × 10^5^ cells/well) were exposed for 24 h to equal volumes of fresh complete DMEM or RPMI 1640 and the filtered viral supernatant, supplemented with 10 μg/mL polybrene (10 mg/mL sterile solution) (Sigma-Aldrich, TR-1003, Burlington, MA, USA). Stable populations were selected using puromycin dihydrochloride (Sigma-Aldrich, P9620). For BT549 cells, 4 μg/mL puromycin was used for selection, while 2 μg/mL was used for other cell lines. Overexpression (OE) and knockdown (KD) efficiency were validated by RT-qPCR and western blotting.

**Table 1:** The sequences of shRNA targets.

Identifier	Forward (5′-3′)
*SNX9*-shRNA#1	GCTAACACCTACTAACACTAA
*SNX9*-shRNA#2	GATGGAATGTAATCACGAGTA
*SNX9*-shRNA#3	GCTGCTGAACCTGGAAATAAT

### 2.3 Single-Cell-Derived Clone Isolation and Cell Morphological Analysis

To isolate single-cell-derived MDA-MB-231 SNX9-knockdown clones, a serial limiting-dilution method was applied. Briefly, a starting suspension of 1000 cells was serially diluted tenfold and distributed into 96-well plates at an estimated density of one cell/well. After seeding, the wells were examined under an inverted microscope, and wells containing a single visually identifiable cell were marked and monitored. Colonies derived from individual cells were allowed to expand and were subsequently transferred to larger culture vessels for further experiments.

Phase contrast images of BC cells were obtained with an Olympus BX43 microscope (bright-field mode; Olympus Corporation, Hachioji, Tokyo, Japan). After calibrating each image to its scale bar, ImageJ v1.54g (NIH, Bethesda, MD, USA) was used to measure the maximum end-to-end distance along the major axis of each cell by using the straight-line tool. Only individual cells with clearly distinguishable boundaries and without substantial overlap with neighboring cells were included in the analysis. For every group, 10 cells were measured across five randomly chosen fields in each of three independent experiments.

### 2.4 Cell Viability Assay

Cell growth kinetics were monitored with the CellTiter AQueous One Solution MTS (Promega, G3580, USA) assay. MDA-MB-231 and MCF-7 cells were plated at 1000 cells/well and BT549 cells at 500 cells/well in 0.1 mL complete medium in 96-well plates, with 3–6 technical replicates for each condition. The plates were cultured for 4–6 days, and cell viability was assessed daily or every other day. At each time point, 10 μL/well MTS reagent was added. Plates with MDA-MB-231 and MCF-7 were incubated for 2 h, and those with BT549 for 1.5 h at 37°C. A CLARIOstar plate reader (BMG Labtech, Germany) recorded absorbance at 490 and 630 nm under an absorbance end-point readout mode, and the background-corrected absorbance was calculated as A_490nm_ − A_630nm_. Proliferation curves were generated from Prism Version 9.0 (GraphPad, San Diego, CA, USA). Values are normalized to the relevant control, and groups are compared at each time point. Results are shown as mean ± SD.

### 2.5 Bromodeoxyuridine (BrdU) Incorporation Assay

The BrdU colorimetric kit (Cell Proliferation BrdU ELISA, No. #11647229001, Roche, Switzerland) was used to determine the cell proliferation rate. MCF-7 and MDA-MB-231 cells were seeded at 2000 cells/well and BT-549 cells at 1000 cells/96-well in 0.1 mL medium and put in the incubator for 12–16 h’s attachment. Each condition included at least three technical wells. Cells received 10 μL/well of the BrdU labeling solution (provided in the kit) for 4 h. The OD value measured immediately after this initial labeling was considered the baseline (day 1), and the subsequent steps, including washing, secondary antibody incubation, and reaction after adding substrate solution, were performed as indicated in the manufacturer’s documentation. The same BrdU labelling, washing, and detection protocol was repeated on day 4. Specifically, in accordance with the no-stop-solution procedure (Step 10 of the kit instructions), the optical density was recorded at both 370 nm and 492 nm (as reference) on a plate reader (CLARIOstar, BMG Labtech, Germany) under an absorbance end-point readout mode. At least two independent biological experiments were carried out.

### 2.6 Colony Formation Assay

To assess cell growth potential, a colony formation assay of BT549, MCF-7 and MDA-MB-231 cells was performed. Cells were plated at 500–1000 cells per well of six-well plates, with at least three wells per condition. Selective complete medium was renewed every 3 days. Cultures were maintained for 10–15 days and terminated when control colonies remained distinct and had not extensively merged (approximately 10 days for 500/well MDA-MB-231 cells). Colonies were fixed with 4% paraformaldehyde (PFA) (Beyotime, P0099-500ml, Shanghai, China) for 30 min at laboratory temperature, and stained overnight using 1% (w/v) crystal violet in methanol (Sigma-Aldrich, C0775). After gentle PBS washing and air drying, plates were photographed using a smartphone in document-scanning mode.

### 2.7 Wound Healing Assay

Wound closure was measured in BT-549 and MDA-MB-231 monolayers. Cells were distributed at 3 × 10^5^ BT-549 or 2 × 10^5^ MDA-MB-231 cells/12-well, with at least three replicate wells, and cultured overnight until reaching near 100% confluence. A linear gap was created with a sterile 200 μL pipette tip, and the medium was changed to fresh medium containing 1% FBS to limit proliferation while avoiding the loss of viability associated with serum deprivation. The wounds were then photographed under an Olympus BX43 microscope (bright-field mode) at 0, 24, and 48 h. The areas of the scratches were calculated using ImageJ v1.54g, and normalized to the average area at 0 h. The experiment was conducted with a minimum of three independent assays performed.

### 2.8 Transwell Assay

Cell 3D migration and invasion were quantified in Transwell inserts (8.0-μm pore size; Corning, 3422, NY, USA). For the migration assay, the top chamber remained uncoated. For invasion experiments, the upper compartment was precoated with Matrigel matrix (Corning, 354230). Briefly, the Matrigel was aliquoted and stored at −80°C. One day before the experiment, the Matrigel aliquot was thawed for several hours at 4°C. On the experimental day, the Matrigel was kept on ice and quickly diluted 1:10 in chilled serum-free medium and applied to the upper well at 100 μL/insert for 2 h at 37°C. During incubation time, prepare the cells for seeding. Cells were suspended in 100 μL serum-free medium and added to the upper compartment. Migration assays used 5 × 10^4^ MDA-MB-231, 8 × 10^4^ MCF-7, or 5 × 10^4^ BT-549/well; invasion assays used 1 × 10^5^ MDA-MB-231, 1.5 × 10^5^ MCF-7, and 1 × 10^5^ BT-549/well. The lower chamber contained 300 μL medium containing 10% FBS. After culturing for 48 h (for migration) or 72 h (for invasion), non-migrated cells were gently removed from the upper surface with a cotton swab. Cells on the lower chamber side were fixed with 4% PFA for 30 min, and stained with 1% crystal violet (Sigma-Aldrich, C0775) for 2 h at ambient temperature. Representative fields were imaged with an Olympus BX43 microscope (bright-field mode) under a 10× objective, and cells were counted manually in five random fields under the 20× objective.

### 2.9 Flow Cytometry Analysis

Cell-cycle distributions of transfected cells were measured by propidium iodide (PI) staining with flow cytometry. MCF-7 (3 × 10^5^ cells/well) and MDA-MB-231 (2 × 10^5^ cells/well) were plated in six-well plates. After overnight incubation for attachment, cultures were rinsed and serum-starved for 24 h to synchronize their cycle. To induce cell cycle re-entry, the medium was returned to medium containing 10% FBS (Thermo Fisher Scientific, 10270106) for a further 24 h. Trypsinized cells were adjusted to 2 × 10^6^ cells/mL in cold PBS and fixed with 70% ice-cold ethanol. Fixed cells were washed by centrifugation in cold PBS (500× *g*, 5 min each, 3 times, 4°C), resuspended in 3 mL of cold PBS, transferred to a flow tube and pelleted once more. PI/RNase staining buffer (BD Biosciences, 550825, San Jose, CA, USA) was applied for 15–30 min at 22~25°C. Samples were detected on a NovoCyte Advanteon BVYG Flow Cytometer (Agilent Technologies, Santa Clara, USA), and data were analyzed by FlowJo v7.6 (FlowJo LLC, Ashland, USA).

### 2.10 Hematoxylin & Eosin (H&E) Staining

Xenografted tumors and lungs were first soaked in 4% PFA fixation buffer for more than 48 h. The tissues were washed in water, slowly dehydrated in a gradient ethanol solution, and then cleared in xylene. After overnight paraffin infiltration, specimens were embedded with fresh paraffin and cooled at 4°C. To ensure section adhesion, glass slides were pre-coated with 3-aminopropyltriethoxysilane (Sigma-Aldrich, A3648), and 4-μm sections were made with a Leica RM2125 rotary microtome (Leica Biosystems, Nussloch, Germany). Sections were dewaxed, rehydrated, stained sequentially with hematoxylin and eosin, dehydrated, and mounted with Canada balsam (Sigma-Aldrich, 60610). Dried slides were imaged in bright-field mode with an Olympus BX43 microscope.

### 2.11 Reverse Transcription Quantitative Polymerase Chain Reaction (RT-qPCR)

Total RNA was extracted with RNAiso Plus solution (TAKARA #9109, Takara Bio Inc., Kusatsu, Shiga Prefecture, Japan), and RNA quality was checked from the A_260_/A_280_ ratio. One microgram of total RNA served as template for cDNA synthesis within a 10-μL reaction mix using a reverse transcription kit (TAKARA #RR047Q): incubating 15 min at 37°C, then incubating 5 s at 85°C. The cDNA samples underwent tenfold dilution in nuclease-free water (Thermo Fisher Scientific, AM9932) to 10 ng/μL. Primer sequences are given in Table 2. Each 10-μL RT-qPCR reaction contained 5 μL of 2× SYBR Green PCR Master Mix (TAKARA #RR820A), 4 μL of 1-μM forward/reverse primer mix solution (0.4 μM final concentration), and 1 μL of template cDNA (10 ng/μL). Reactions were run on a Roche LightCycler 480 II instrument (Roche, Basel, Switzerland) with an initial denaturation at 95°C for 0.5 min (Stage I, 1 cycle); 40 cycles (Stage II) of denaturation at 95°C for 0.08 min and annealing/extension at 61°C for 0.5 min; followed by a final hold at 4°C (Stage IV, 1 cycle). Employing the 2^-ΔΔCt^ method, with glyceraldehyde-3-phosphate dehydrogenase (*GAPDH)* as the reference, we quantified the relative expression of target genes. Each target was analyzed in at least three biological samples with three technical replicates.

**Table 2:** Primer sequences of genes in the RT-qPCR analysis.

Target Gene	Primers (5′-3′)
*SNX9*-Forward primer	ATGGCCCAATGTGGGTTTATC
*SNX9*-Reverse primer	AGGAGACGCTCATATAACCAGTC
*GAPDH*-Forward primer	ACCCAGAAGACTGTGGATGG
*GAPDH*-Reverse primer	TTCAGCTCAGGGATGACCTT

### 2.12 Western Blotting

Protein was quantified by BCA assay (Beyotime, P0012), and 10–20 μg per channel were resolved by SDS-PAGE gel according to target abundance. An 8% gel was used for target proteins larger than 100 kDa, while 10% and 12% gels were used for other target proteins. Following wet transfer to PVDF membranes (Bio-Rad, 1620177, Hercules, CA, USA), membranes were placed in 3–5% bovine serum albumin (BSA) (Sigma-Aldrich, A5611) for 1 h under room conditions, and then overnight exposed to the primary antibody solution (Table 3) at 4°C. After TBS-T washes, the appropriate HRP-conjugated secondary antibody (Table 3) was applied for 45–90 min at room temperature. Bands were developed with Immobilon chemiluminescent substrate (Millipore, WBKLS0500, Burlington, MA, USA) and captured on a Bio-Rad ChemiDoc imaging system. Densitometry was performed in ImageJ Version 1.54g, using GAPDH, β-tubulin, and β-actin as normalization standards for sample loading. At least two independent biological replicates were performed.

**Table 3:** Antibodies list and details.

Antibody Name	Catalog No.	Dilution	Company/City, Province/State, Country
SNX9	ab181856	1:2000	Abcam/Cambridge, Cambridgeshire, UK
PCNA	ym3031	1:4000	ImmunoWay/San Jose, California, USA
E2F1	A2067	1:1000	Abclonal/Woburn, Massachusetts, USA
E2F6	A2718	1:1000	Abclonal
CDK6	#3136	1:1000	Cell Signaling Technology (CST)/Danvers, USA
CDK2	A0094	1:1000	Abclonal
RB	#9309	1:1000	CST
p-RB (Ser807/811)	#9308	1:500	CST
p27	#2552	1:1000	CST
MMP9	A2095	1:1000	Abclonal
TKS5	A9363	1:1000	Abclonal
EGFR	A11351	1:1000	Abclonal
p-EGFR (Y1068)	AP0027	1:500	Abclonal
p-EGFR (Y1173)	AP0992	1:500	Abclonal
MEK 1/2	#9122	1:1000	CST
p-MEK 1/2	#9154	1:1000	CST
ERK 1/2	#4695	1:1000	CST
p-ERK 1/2	#4370	1:1000	CST
Pan-Akt	#4691	1:1000	CST
p-Akt	#9271	1:500	CST
β-tubulin	AC021	1:2000	Abclonal
GAPDH	AC002	1:2000	Abclonal
β-actin	#4970	1:5000	CST
HRP-conjugated Anti-Rabbit secondary antibody	#7074	1:5000	CST
HRP-conjugated Anti-Mouse secondary antibody	#7076	1:5000	CST
**For Immunofluorescence staining**			
PCNA	ym3031	1:500	ImmunoWay
F-actin	C2205S	1:400	Beyotime/Shanghai, China
SNX9	ab181856	1:400	Abcam
TKS5	A9363	1:400	Abclonal
Goat Anti-Rabbit IgG H&L (Alexa Fluor 488)	ab150077	1:500	Abcam
Goat Anti-Rabbit IgG H&L (Alexa Fluor 555)	ab150078	1:500	Abcam
Goat Anti-Mouse IgG H&L (Alexa Fluor 594)	ab150116	1:500	Abcam
**For Immunohistochemistry staining**			
SNX9	ab181856	1:200	Abcam
**For Immunoprecipitation analysis**			
SNX9	ab181856	1:50	Abcam

### 2.13 Immunofluorescence (IF) Imaging

For immunofluorescence, MDA-MB-231 and MCF-7 cells were plated on 0.1% gelatin (Sigma-Aldrich, G1890)-coated coverslips in 24-well plates at 20,000 cells and 40,000 cells per well, respectively. At 60–80% confluence, cells were washed with PBS, fixed in 4% PFA for 30 min at room temperature, and permeabilized for 15 min with 0.1% Triton X-100 in PBS. After blocking with 3% BSA (Sigma-Aldrich, A5611) in PBS, cells were placed in the indicated primary antibody solution (Table 3) overnight in a dark and humidified environment at 4°C. An appropriate fluorophore-conjugated secondary antibody (Table 3) was then applied at 1:500 for 1 h at room temperature. F-actin staining was labelled for 30 min with actin-tracker dye (Beyotime, C2205S; 1:400), and nuclei were counterstained for 15 min at ambient temperature with 1 μg/mL DAPI (Cell Signalling Technology, 4083). Three washes with 0.1% PBS-T (Tween-20) were performed between steps. Images were captured on the LSM 800 confocal microscope (Carl Zeiss AG, Oberkochen, Germany).

### 2.14 Immunohistochemistry (IHC) Analysis

Paraffin-embedded tumor slides were dewaxed in xylene and gradually rehydrated in different concentrations of ethanol/water mixture. For SNX9 staining, slides were heated for 20 min at 95–100°C in Tris-EDTA buffer (pH 9.0) (Beyotime, P0084), and cooled to room temperature in the same solution. After PBS washing, endogenous peroxidase was quenched for 10 min with the reagent from the Histostain™-Plus Kit (ZSGB-BIO, SP-9000, Beijing, China), followed by a 30-min block with BSA solution (from the same kit). Sections were incubated overnight at 4°C with the primary SNX9 antibody (Table 3). Biotinylated goat anti-rabbit IgG polymer and horseradish peroxidase-conjugated streptavidin working solution (from the same kit) were then sequentially incubated for 10–15 min each, with three 3-min PBS washes between steps. Fresh DAB+ substrate (Agilent Technologies, K3467, Dako, Santa Clara, USA) was developed for 0.5–1 min, then stopped with running tap water. Sections were counterstained with hematoxylin, rinsed in running water to blue, and allowed to air-dry briefly. Then, the sections were mounted with an aqueous mounting medium (glycerol). As an aqueous mounting medium was used, dehydration through graded ethanol and xylene clearing were omitted. Slides were immediately imaged with an Olympus BX43 microscope (bright-field mode). No formal scoring was performed; staining was evaluated by three independent observers.

### 2.15 Immunoprecipitation (IP) Assay

The IP assay used the Protein G Immunoprecipitation Kit (Roche, 11719386001). SNX9-OE cells were harvested and solubilized in lysis buffer (from the kit) containing 1× protease and phosphatase inhibitor cocktail (Thermo Fisher Scientific, 78442), and lysates were clarified at 4°C and 15,000× *g* for 30 min. Samples were precleared for 3 h by rotating with Protein-G-agarose beads (from the kit) under refrigeration. Ten percent of each lysate (100 μL) was retained as input; 400-μL aliquots were then incubated for 1 h at 4°C with either 8 μL anti-SNX9 antibody (1:50; Table 3) or 4 μL control immunoglobulin G (Santa Cruz Biotechnology, sc-2027, Dallas, TX, USA). Subsequently, 50 μL protein-G-agarose beads (from the kit) were distributed to each reaction and the mixtures were incubated overnight at 4°C. Beads were washed in cold wash buffer (from the kit) for 5 min per wash and resuspended in 40 μL 2× western loading buffer. After boiling at 100°C for 5–10 min and centrifugation at 1000× *g* for 3 min, 15 μL of each sample (input, IgG control, and SNX9-immunoprecipitated) was resolved on an 8% SDS-PAGE gel, transferred to PVDF, and immunoblotted for tyrosine kinase substrate with five SH3 domains (TKS5) (Table 3).

### 2.16 Rho Guanosine Triphosphatase (RhoGTPase) Pull-Down Activation Assay

To assess the activities of small RhoGTPases, the activation assay kit (Cytoskeleton, BK030, Denver, USA) was utilized. SNX9-manipulated cells were lysed in cell lysis buffer with 1× protease inhibitors, and the clarified supernatant was stored at −80°C, with aliquots retained for BCA quantitation and small G-protein immunoblotting. For each pull-down, 500 μg protein (balanced by volume with lysis buffer) was incubated with 10 μg (10 μL) p21-activated Kinase-p21-binding domain (PAK-PBD) beads (supplied in the kit, specifically bind the guanosine triphosphate (GTP)-bound forms of Ras-related C3 botulinum toxin substrate 1 (Rac1) and cell division cycle 42 homolog (Cdc42)) for 1 h at 4°C on a rotator. Beads were collected at 3000× *g* for 3 min and washed three times at 4°C with Wash buffer (provided in the kit). Bound proteins were boiled with 20 μL 2× sample buffer and then resolved with 12% SDS-PAGE gels. After blocking in 5% BSA for 1 h, membranes were immunoblotted with kit-supplied anti-Cdc42 (1:250) and anti-Rac1 (1:500) in TBS-T, with constant shaking overnight at 4°C. The membrane was exposed to anti-mouse secondary antibody for 1 h at 22°C before chemiluminescence detection. Quantified His-tagged protein Cdc42 and Rac1 (provided in the kit) were run in parallel as positive controls.

### 2.17 Animal Studies

Experiments involving animals were authorized by the Licensing Committee on the Use of Live Animals in Teaching and Research (CULATR No. 4483-17, 4484-17, 5420-20, and 5161-19) of the University of Hong Kong. A total of around 20 female BALB/cAnN-nu nude mice (4–6 weeks of age, 18–22 g upon arrival) were supplied by the university’s Centre for Comparative Medicine Research. Animals were housed in IVC cages in a specific-pathogen-free environment with a 12-h light-dark photoperiod, a temperature of 22 ± 2°C, controlled humidity, and unrestricted access to sterilized food and water. Following 1 week of acclimatization, mice were randomly allocated to the predefined groups. Animal allocation and experimental endpoints were specified prior to the commencement of the study.

For the subcutaneous xenograft model, each mouse received SNX9-OE MCF-7 cells in the left axillary mammary fat pad, and vector-control MCF-7 cells in the right pad (n = 3 mice, each mouse serving as its own bilateral control). For the SNX9 knockdown experiment, shSNX9 MDA-MB-231 cells were injected into the left pad, and shNC cells were injected into the right pad in a separate cohort of 3 mice. Cell suspensions were prepared in sterile PBS at 1 × 10^7^ cells/mL, and 100 μL was delivered at each site. Tumors and body weights were measured twice weekly. Volume was calculated as width^2^ × length × 0.5. After approximately 4 weeks, animals were euthanized and tumors excised and photographed. Each tumor was divided, with one half fixed in 4% PFA and the other snap-frozen in liquid nitrogen.

Experimental metastasis was modeled in the lung and bone, two frequent sites of BC dissemination. For the lung metastasis model, MDA-MB-231 cells (shNC or shSNX9#2; 1 × 10^6^ cells in 100 μL PBS) were delivered through the lateral tail vein (n = 3/group). Lung colonization was monitored weekly with a PE IVIS Spectrum system (PerkinElmer, Waltham, MA, USA). For imaging, mice received intraperitoneally injection of D-luciferin (150 mg/kg; Gold Biotechnology, LUCK-100, St. Louis, MO, USA), were anesthetized with isoflurane, and were imaged 5 min later. Mice were sacrificed 5 weeks after inoculation, lung tissues were excised, embedded in paraffin, and examined by H & E staining.

For the bone metastasis model [25], a total of 6 × 10^5^ MDA-MB-231 cells (shNC or shSNX9#2) in 100 μL PBS were carefully injected into the left cardiac ventricle of nude mice (n = 5/group). Successful injection was confirmed by immediate bioluminescence imaging. The health status of all mice was monitored daily and scored according to a pre-defined humane endpoint scoring sheet, which included paralysis, hunched posture, dehydration, weight loss, inactivity, abnormal breathing, low body temperature, eye diseases, and visible tumors. Each parameter was scored as 0 (absent), 1 (moderate), or 2 (severe). Mice accumulating a total score ≥ 8 were euthanized immediately. Bioluminescence imaging was performed weekly as described above. Surviving mice were sacrificed 2 months after inoculation, and hind-limb bones were collected and preserved in 4% PFA or liquid nitrogen.

### 2.18 Bioinformatics Analysis

The prognostic relationship between SNX9 and BC outcome was assessed using publicly available online survival analysis platforms. Transcriptome sequencing datasets and corresponding clinical parameters for breast invasive carcinoma were derived from The Cancer Genome Atlas (TCGA) database (Project ID: TCGA-BRCA). In UALCAN (http://ualcan.path.uab.edu) [26], the “Breast invasive carcinoma” expression dataset was selected, cases were divided into high- and low-expression groups based on the median transcripts-per-million value, and overall survival was compared by log-rank testing. In GEPIA2 (http://gepia2.cancer-pku.cn), the “Overall Survival” analysis method was used with the group cutoff set to “Median”, 95% confidence intervals displayed, and the dataset limited to “BRCA”. The association was also evaluated with the breast cancer cohort in the Kaplan-Meier plotter (https://kmplot.com) using the default “Auto select best cutoff”. All survival curves were generated directly by the respective web tools without additional data processing.

### 2.19 Data Analysis

Analyses were conducted in GraphPad Prism 9.0 software. Student’s *t*-test was applied to two-group comparisons. Experiments involving ≥3 groups were analyzed via one-way analysis of variance (*ANOVA*) and Dunnett’s multiple comparisons test. Distributional normality was checked via the Shapiro-Wilk test. Equality of variance was examined with the F test for two-group comparisons and Brown-Forsythe and Bartlett’s tests for multiple groups. Results were reported as mean ± standard deviation (SD), with *p* values < 0.05 considered statistically significant. Significance relative to the corresponding control is denoted as *p < 0.05, **p < 0.01, ***p < 0.001, and ****p < 0.0001.

## 3 Results

### 3.1 Overexpressed SNX9 Expression Links to Unfavorable Survival Outcomes among BC Patients

**Figure 1:**
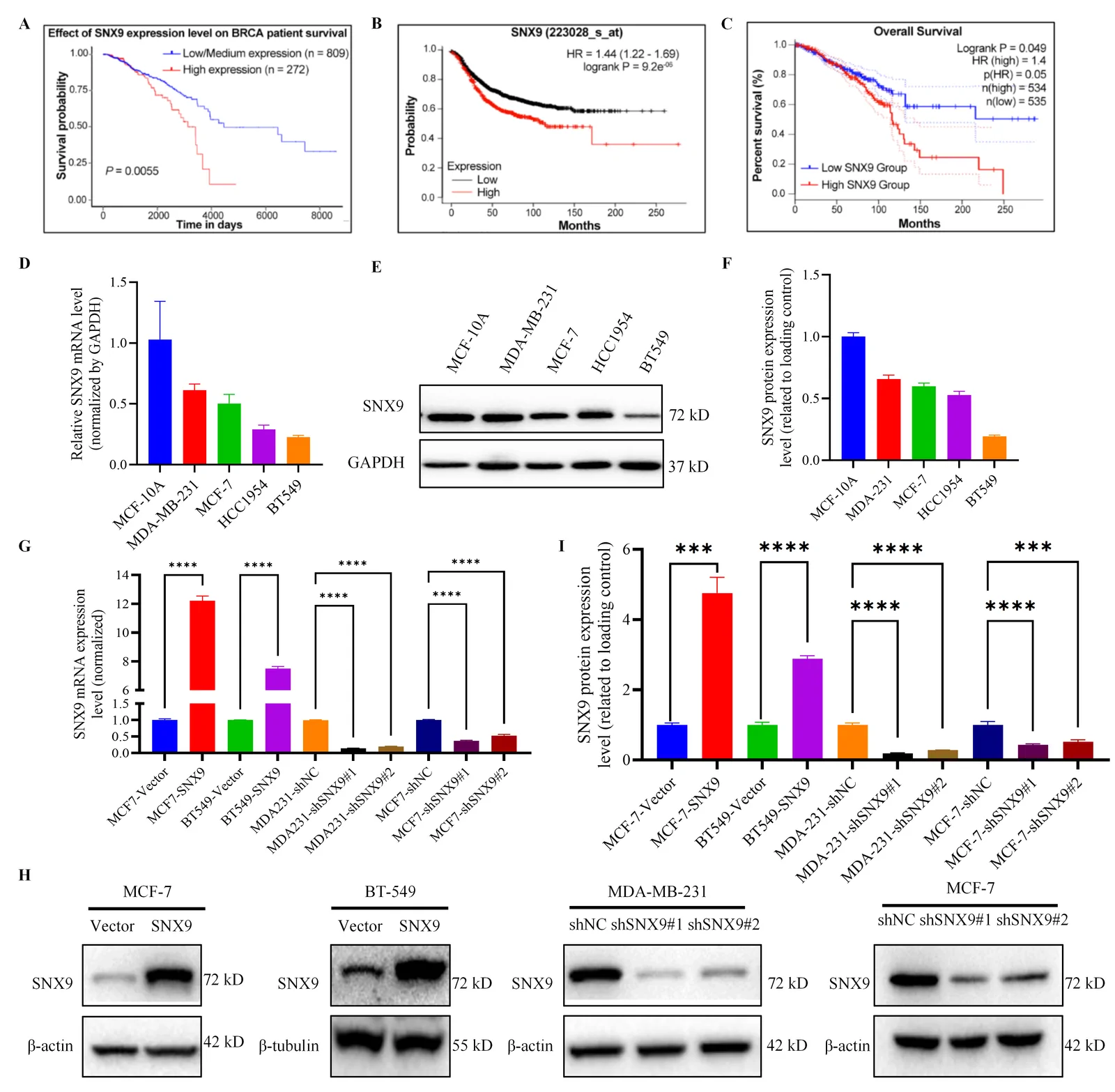
**Higher expression level of SNX9 predicts a shorter overall survival time for BC patients.** (**A**) Survival curve of the TCGA breast cancer cohort using UALCAN. Corresponding analyses were performed with (**B**) Kaplan-Meier and (**C**) GEPIA2. The expression level of SNX9 in BC cell lines and MCF-10A cells was measured by (**D**) RT-qPCR assay and (**E**) western blot analysis; (**F**) densitometric quantification of the western blots. SNX9 protein was normalized to GAPDH and expressed relative to MCF-10A, which was set as 1.0. SNX9 expression levels in overexpressed and knockdown cells were validated by (**G**) RT-qPCR and (**H**) western blot assays; (**I**) quantitative analysis of western blots. Two-group comparisons used Student’s *t*-test, whereas experiments with more than two groups used one-way *ANOVA*. Values are mean ± SD, ***p < 0.001, ****p < 0.0001 versus the corresponding control. Abbreviations: SNX9, sorting nexin 9; BRCA, breast cancer; HR, hazard ratio; GAPDH, glyceraldehyde-3-phosphate dehydrogenase.

We first tested whether SNX9 expression carries prognostic information in BC. The analysis of the TCGA-BRCA cohort through UALCAN [26] associated higher SNX9 expression with reduced overall survival (Fig. 1A). Independent interrogation with the Kaplan-Meier plotter and GEPIA2 databases produced the same direction of association (Fig. 1B,C).

### 3.2 SNX9 Promotes BC Cell Proliferation In Vitro and Accelerates Tumor Expansion In Vivo

SNX9 transcript and protein abundance were next compared among several BC cell lines and the non-tumorigenic mammary epithelial line MCF-10A by RT-qPCR (Fig. 1D) and western blotting (Fig. 1E,F), respectively. Based on the relative SNX9 abundance, MCF-7, BT-549, as well as MDA-MB-231 BC cells were selected for functional studies. We then generated SNX9-overexpressing (SNX9) or control (Vector) BC cells via lentiviral transduction in MCF-7 and BT-549 cells, and stable SNX9-silenced (shSNX9#1 and shSNX9#2) or negative control (shNC) BC cells using lentiviral particles in MCF-7 and MDA-MB-231 cells. RT-qPCR (Fig. 1G) and western blotting (Fig. 1H,I) verified the intended changes in SNX9 expression.

**Figure 2:**
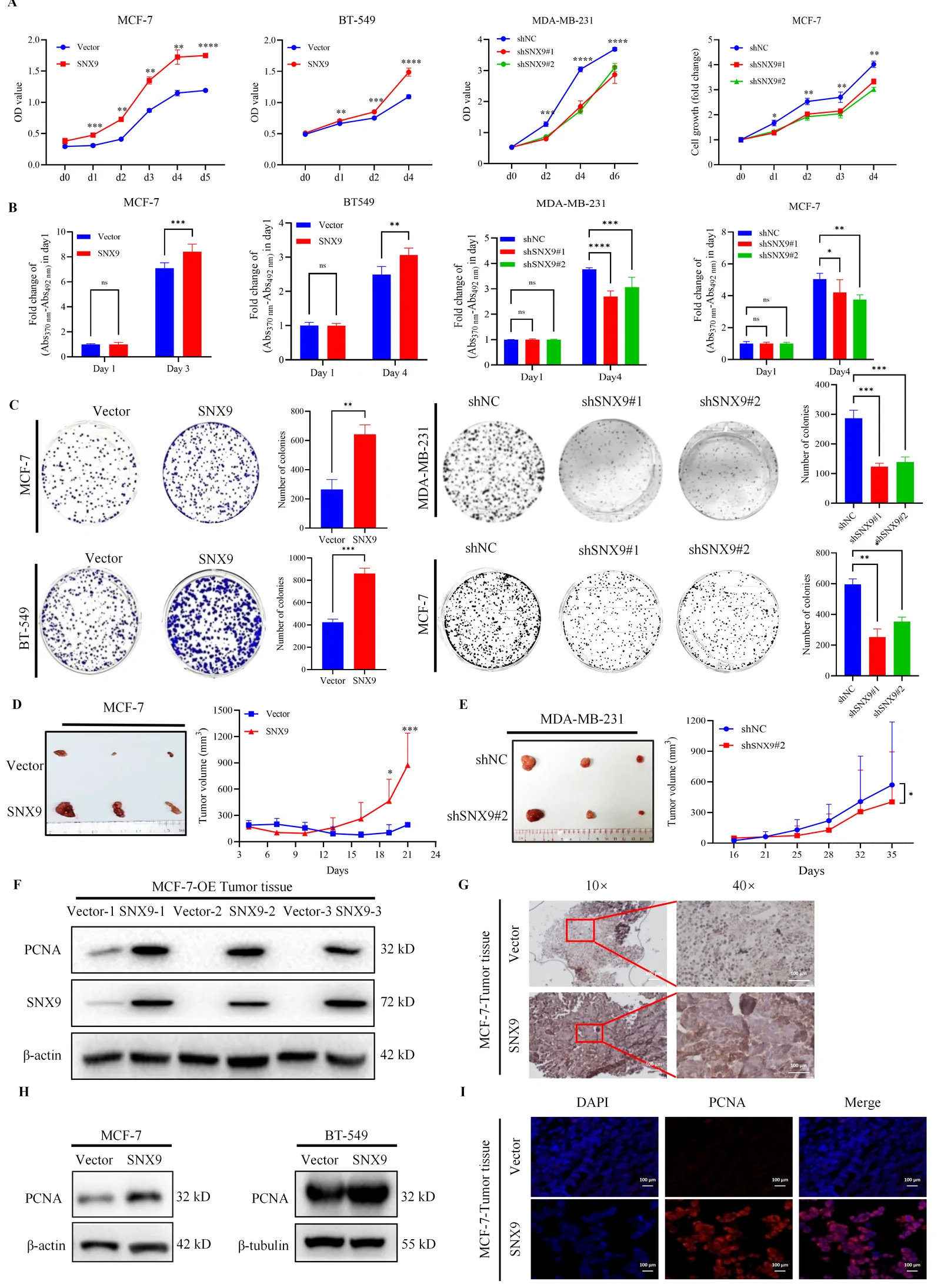
**Promoting effects of SNX9 on breast carcinoma cell proliferation and xenograft growth.** (**A**–**C**) Proliferation after SNX9 OE or KD in cells was evaluated by (**A**) MTS, (**B**) BrdU incorporation, and (**C**) colony formation assays. (**D**,**E**) *In vivo* tumor xenograft growth was assessed with (**D**) SNX9 OE on MCF-7 cells and (**E**) SNX9-KD MDA-MB-231 cells. (**F**) The expression of SNX9 and PCNA in SNX9-OE MCF-7 tumor tissues was assessed by Western blot assay. (**G**) Immunohistochemical detection of SNX9 in SNX9-OE MCF-7 tumor tissues (Scale bar: 100 μm). The effect of SNX9 OE on PCNA expression was evaluated by (**H**) Western blot assay in SNX9-OE MCF-7 and BT-549 cells and (**I**) IF assays in SNX9-OE MCF-7 tumor tissues (Scale bar: 100 μm). Data were analyzed by Student’s *t*-test for two-group comparisons (**A**–**E**), and one-way *ANOVA* (**A**–**C**) was used for comparisons involving over two groups. Data are mean ± SD. *p < 0.05, **p < 0.01, and ***p < 0.001 and ****p < 0.0001 vs. the corresponding control; ns: not significant. Abbreviations: SNX9, sorting nexin 9; OD, optical density; PCNA, proliferating cell nuclear antigen.

To evaluate the tumorigenic potential of SNX9, we conducted several *in vitro* functional assays, including the MTS assay, colony formation assay, and BrdU incorporation assay, to examine cell growth. SNX9-overexpressing (SNX9) cells exhibited significantly increased proliferation rates (Fig. 2A), higher BrdU incorporation abilities (Fig. 2B), and enhanced colony-forming frequencies (Fig. 2C) compared to vector groups. Conversely, KD of SNX9 expression showed the opposite effect (Fig. 2A–C). We then tested these findings in xenograft models using SNX9-overexpressing MCF-7 cells or SNX9-depleted MDA-MB-231 cells and their matched controls. SNX9 OE increased xenograft volume (Fig. 2D), whereas the inverse trend was noted in SNX9-depleted cells (Fig. 2E). The expression of SNX9 in the SNX9-OE MCF-7 tumor samples was determined by Western Blot (Fig. 2F) and IHC (Fig. 2G) assays. Proliferating cell nuclear antigen (PCNA), a DNA-replication-associated proliferation marker [27], was not only significantly increased in SNX9-OE MCF-7 and BT-549 cells (Fig. 2H) but also upregulated in SNX9-OE MCF-7 tumor samples determined by Western Blot (Fig. 2F) and IF assay (Fig. 2I), indicating the tumor-promoting ability of SNX9 in BC.

### 3.3 Silencing of SNX9 Arrests the Cell Cycle at the G2/M Checkpoint in BC Cells

**Figure 3:**
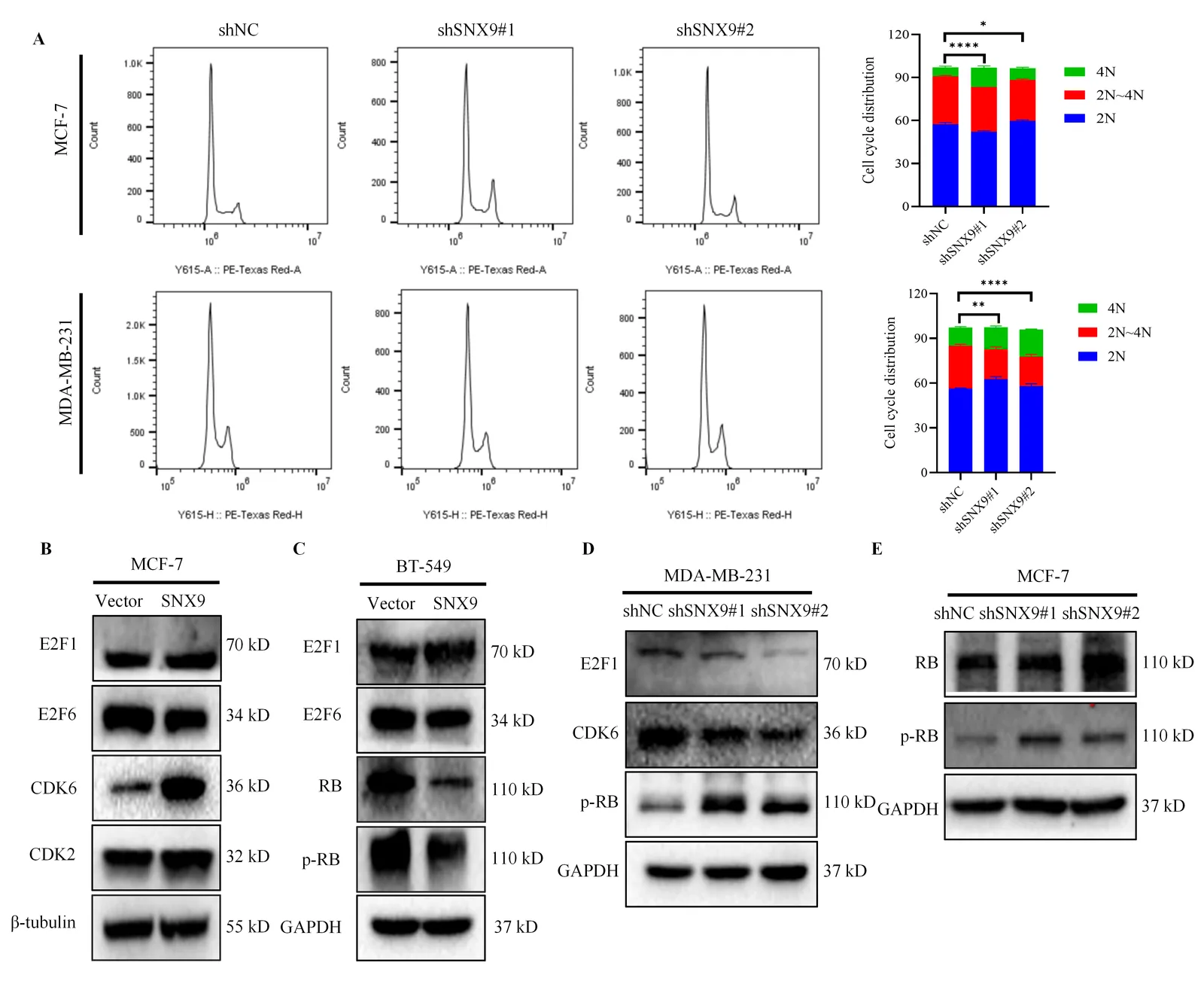
**Silencing of SNX9 increases the G2/M fraction of breast cancer cells.** (**A**) Cell cycle distribution of SNX9-KD MCF-7 and MDA-MB-231 cells was measured by flow cytometry. Related cell cycle markers were investigated by WB in (**B**) SNX9-OE MCF-7 cells, (**C**) SNX9-OE BT-549 cells, (**D**) SNX9-KD MDA-MB-231 cells, and (**E**) SNX9-KD MCF-7 cells. Panel A was analyzed by one-way *ANOVA*. The results are mean ± SD. *p < 0.05, **p < 0.01, and ****p < 0.0001 versus the corresponding control. Abbreviations: SNX9, sorting nexin 9; E2F1, the adenoviral early region 2 binding transcription factor 1; E2F6, the adenoviral early region 2 binding transcription factor 6; CDK2, cyclin-dependent kinase 2; CDK6, Cyclin-dependent kinase 6; RB, retinoblastoma, P indicates phosphorylated form.

Cell cycle progression was assessed by flow cytometry to clarify how SNX9 exerts its growth-regulatory effects. After cell cycle synchronization, FBS (10%) was added to resume cell cycle progression. SNX9-KD cells exhibited a marked accumulation at the G2/M checkpoint relative to shNC controls, indicating that silencing of SNX9 could induce G2/M phase arrest in BC (Fig. 3A). Additionally, cell cycle markers were investigated by western blotting (Fig. 3B–E). Cyclin-dependent kinase (CDK) 6, which can form complexes with CDK4 and D-type cyclins to activate the cell cycle in the early G1 phase [28], was upregulated in SNX9-overexpressed MCF-7 cells and downregulated in SNX9-depleted MDA-MB-231 cells. The CDK4/6-cyclin D complexes phosphorylate retinoblastoma (RB) protein in the mid-G1 phase [29], leading to its inactivation and dissociation of RB-the adenoviral early region 2 binding transcription factor (E2F) complexes, releasing the activator E2Fs, such as E2F1, E2F2, or E2F3A, thereby supporting gene expression for the transition from G1 to S phase and DNA synthesis [28,29,30]. Phosphorylated RB was elevated in MDA-MB-231-KD and MCF-7-KD cells, while decreased in BT-549-OE cells. The E2F family of adenoviral early region 2 binding transcription factors has different functions in the cell cycle process [30]. Increased E2F1 expression was observed in MCF-7-OE and BT-549 OE cells but suppressed in MDA-MB-231-KD cells. Cyclin-dependent kinase inhibitor (CDKI) protein p27^kip1^ primarily targets the CDK2/cyclin E complex to inhibit S phase initiation. Enhanced CDK2 was noticed in MCF-7 OE cells. E2F6, a canonical repressor, accumulates during the S phase and attenuates E2F target gene expression in the G2 phase [30]. Reduced E2F6 expression was observed in MCF-7 and BT-549 OE cells. Therefore, SNX9 can regulate different genes, and their downstream effectors contribute to distinct cell cycle phases.

### 3.4 SNX9 Facilitates the Metastatic Potential of BC Cells Both In Vitro and In Vivo

**Figure 4:**
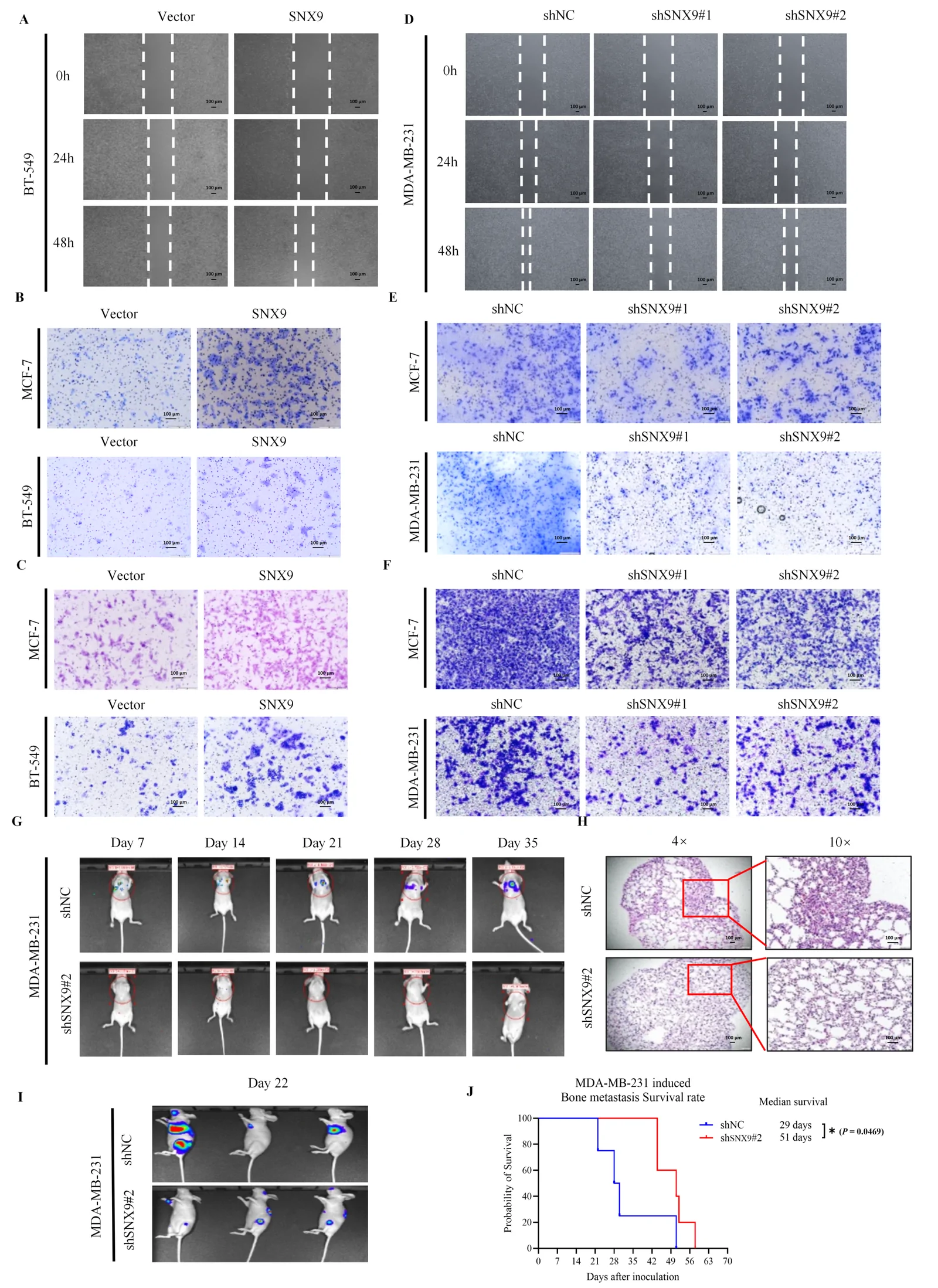
**SNX9 contributes to breast cancer cell motility and metastatic colonization.** (**A**–**F**) Wound healing assay measured 2D migration in (**A**) BT-549 SNX9-OE cells and (**D**) MDA-MB-231 SNX9-KD cells. Uncoated Transwell inserts were utilized to assess 3D migration after SNX9 (**B**) OE or (E) KD, and Matrigel-coated inserts were used to assess invasion after SNX9 (**C**) OE and (F) KD. (Scale bar: 100 μm) (**G**) Bioluminescence in the MDA-MB-231 lung-metastasis model after SNX9 depletion. (**H**) *H&E* analysis of metastatic lung area (Scale bar: 100 μm). (**I**) Bioluminescence in the bone-metastasis model of MDA-MB-231 cells and (**J**) the corresponding survival analysis. Student’s *t*-test was used for the indicated two-group comparisons (Supplementary Fig. S1A–C), and one-way *ANOVA* was used in Supplementary Fig. S1D–F. Survival curves were estimated using the Kaplan-Meier method, and survival in panel J was compared with the Gehan-Breslow-Wilcoxon test. *p < 0.05 vs. the control group. Abbreviations: SNX9, sorting nexin 9.

Previous studies implicated SNX9 in cancer cell migration [22,24]. We therefore compared wound closure and Transwell-based migration and invasion assays after changing SNX9 expression. Transfection with SNX9 resulted in an enhanced wound healing rate in BT-549, cell 3D migration, and Matrigel invasion abilities in MCF-7 and BT-549 (Fig. 4A–C and supplementary Fig. S1A–C) when compared to the vector control. Conversely, when endogenous SNX9 was interfered with by shRNAs, cell motility was significantly reduced (Fig. 4D–F; supplementary Fig. S1D–F).

The effect of SNX9 depletion on metastatic colonization was then evaluated *in vivo*, we inoculated MDA-MB-231-shSNX9#2 or negative control cells through the tail vein and left ventricle of mice to establish lung and bone metastasis models, respectively. In the lung experiment, bioluminescence increased progressively from days 7 to 35 in the control group mice, while remained lower intensity after SNX9 knockdown (Fig. 4G). Histological examination of lung tissues indicated a smaller metastatic tumor area in lungs from the shSNX9#2 group than that in the shNC mice (Fig. 4H). In the intracardiac model, control animals displayed stronger bioluminescent signals by day 22, whereas SNX9 depletion prolonged overall survival time (Fig. 4I,J). Collectively, our data establish SNX9 as a critical driver of BC metastasis, as validated by both cell-based and animal model systems.

### 3.5 SNX9 Modulates Actin Cytoskeleton Remodelling, Cell Morphology Changes, and Pseudopodia Formation in BC Cells

**Figure 5:**
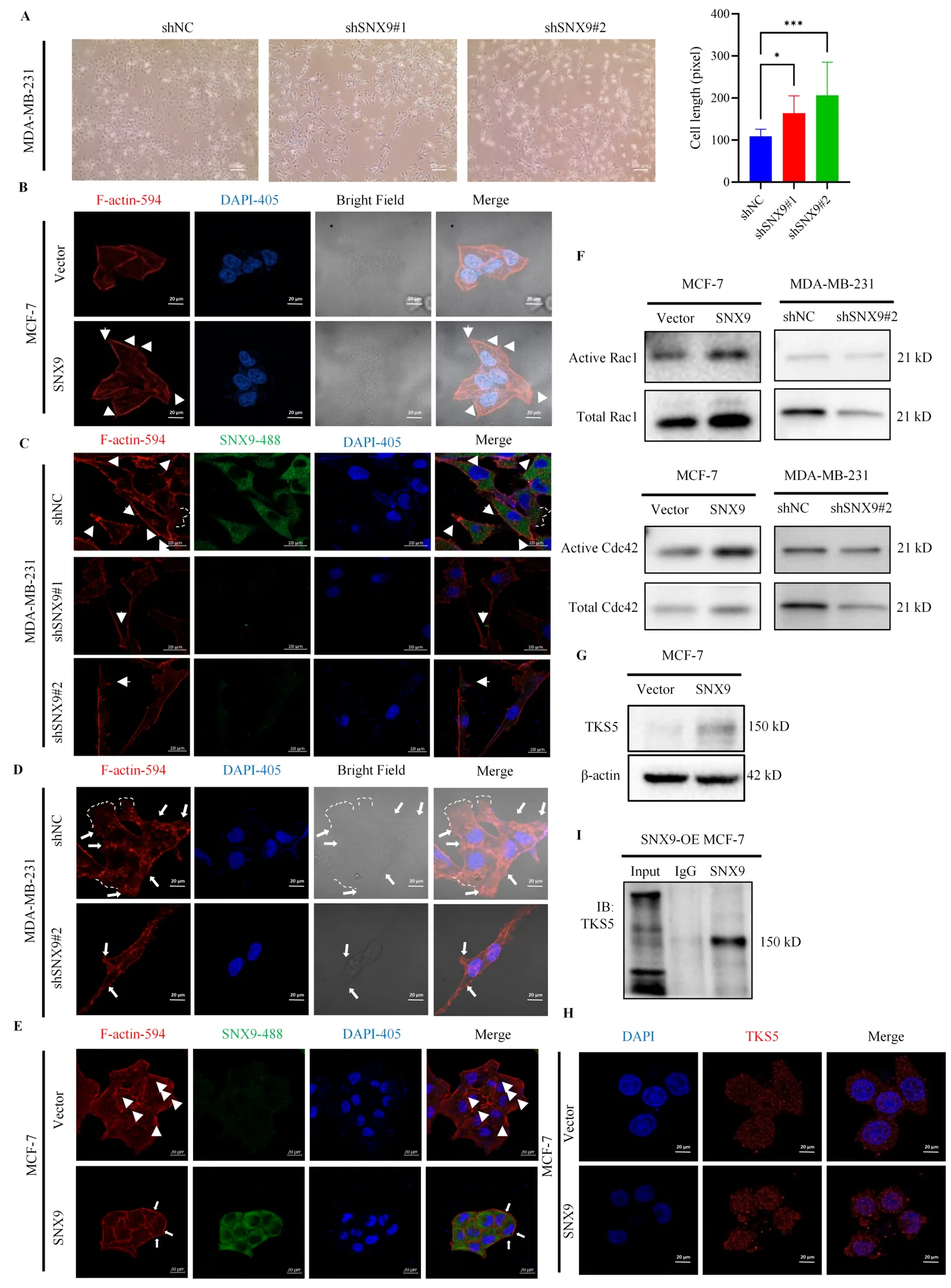
**SNX9 modulates actin cytoskeleton remodeling, leading to changes in cell morphology and promoting pseudopodia formation in breast cancer cells.** (**A**) Bright-field images (Scale bar: 100 μm) and cell-length quantification of the elongated symmetrical spindle-shaped cell morphology observed after SNX9 KD in MDA-MB-231 cells. (**B**) Numerous long filopodia were observed in MCF-7-OE cells. Filopodia (thin arrows) were as indicated. (**C**) Fewer long filopodia were observed in MDA-MB-231 SNX9-KD cells. Filopodia (thin arrows) and lamellipodia (Dotted line) were as indicated. (**D**) Reduced number of lamellipodia was observed after SNX9 depletion in MDA-MB-231 cells. Membrane protrusions (Arrows) and lamellipodia (Dotted line) were as indicated. (**E**) Enhanced actin remodeling and formation of membrane ruffles in MCF-7-OE cells. Stress fibers (Arrowheads) and membrane ruffles (Arrows) were as indicated. (**B**–**E**) Red: F-actin staining; green: anti-SNX9; blue: DAPI. (Scale bar: 20 μm) (**F**) Immunoblot assay of total and active forms of Cdc42 and Rac1 in SNX9-manipulated cells. TKS5 was assessed in MCF-7-OE cells by (**G**) western blotting and (**H**) confocal microscopy (red: TKS5; blue: DAPI). (Scale bar: 20 μm) (**I**) Co-immunoprecipitation of SNX9-associated TKS5. Panel A was analyzed by one-way *ANOVA*. Results are shown as mean ± SD. *p < 0.05, ***p < 0.001 versus the control group. Abbreviations: SNX9, sorting nexin 9; Rac1, Ras-related C3 botulinum toxin substrate 1; Cdc42, cell division control protein 42 homolog; GTP, guanosine triphosphate; TKS5, tyrosine kinase substrate with five SH3 domains.

The actin cytoskeleton undergoes dynamic changes in response to extracellular signals and to facilitate cell movement and endocytosis. To examine whether SNX9 influences cell shape, single-cell-derived MDA-MB-231 knockdown clones were isolated by limiting dilution. Clones with stronger SNX9 depletion displayed a marked increase in cell length, resulting in a symmetrical spindle-shaped morphology. Bright fields of MDA-MB-231 cells showed cell elongation in SNX9-silencing groups based on quantitative analysis (Fig. 5A). The cells became longer and thinner, especially at sub-confluence sites. These morphological alterations likely contribute to impaired cellular motility.

As a key aspect of cytoskeletal biology, filopodia biogenesis has inspired a number of recent studies addressing the assembly machinery that drives filopodia formation [31,32,33]. In MCF-7-SNX9-OE cells, we observed a higher number of long filopodia (thin arrow) than the vector controls (Fig. 5B). Conversely, SNX9-depleted MDA-MB-231 cells exhibited only a few long filopodia at the tip of the tentacle (Fig. 5C). Elongated cell filopodia (upper plane, thin arrow) along with their cell body were seen in shNC cells. The staining of F-actin revealed a conspicuous decrease in the number of membrane protrusions and lamellipodia area (dotted line) in MDA-MB-231 SNX9 KD cells versus shNC cells (Fig. 5D).

MCF-7 cells transfected with a control plasmid displayed a polygonal cell shape with well-organized stress fibers (arrowheads) and formed a complete cell colony with unclear boundaries between cells (Fig. 5E). SNX9 OE cells experienced actin remodelling [34], resulting in the formation of membrane ruffles with their net-like actin, resulting in bulbous, short, and blunt forms (Fig. 5E). This cellular event parallels the epithelial-mesenchymal transition, during which cell-cell attachment is reduced.

Additionally, these results suggested a potential link between activated Rho-GTPases and SNX9-induced actin reorganization, as Rho-GTPase signalling is integral to cytoskeletal dynamics [35]. Rac1 and Cdc42 activity was measured with PAK-PBD pull-down assays. SNX9-overexpressing MCF-7 cells contained more total and GTP-bound Rac1 and Cdc42 than vector controls, whereas both pools were reduced in SNX9-depleted MDA-MB-231 cells (Fig. 5F). The association between SNX9 abundance and Rac1/Cdc42 activity provides a plausible link to the observed cytoskeletal phenotypes, although further investigation is necessary to determine how this occurs and its downstream effectors.

Cell invasion involves the concentration of matrix metalloproteinases to degrade the extracellular matrix (ECM), with invadopodium formation facilitating actin-based invasive protrusions during early stages of three-dimensional invasion. To confirm SNX9-mediated actin remodelling in invadopodia, the abundance of TKS5, an established invadopodial scaffold [36], was increased in MCF-7-SNX9-OE cells by immunoblot and stronger TKS5 staining by confocal microscopy (Fig. 5G,H). Additionally, co-immunoprecipitation detected TKS5 in SNX9-containing complexes (Fig. 5I), supporting an association between the two proteins.

### 3.6 SNX9 Enhances the EGFR/ERK1/2 Signalling Pathway in BC

**Figure 6:**
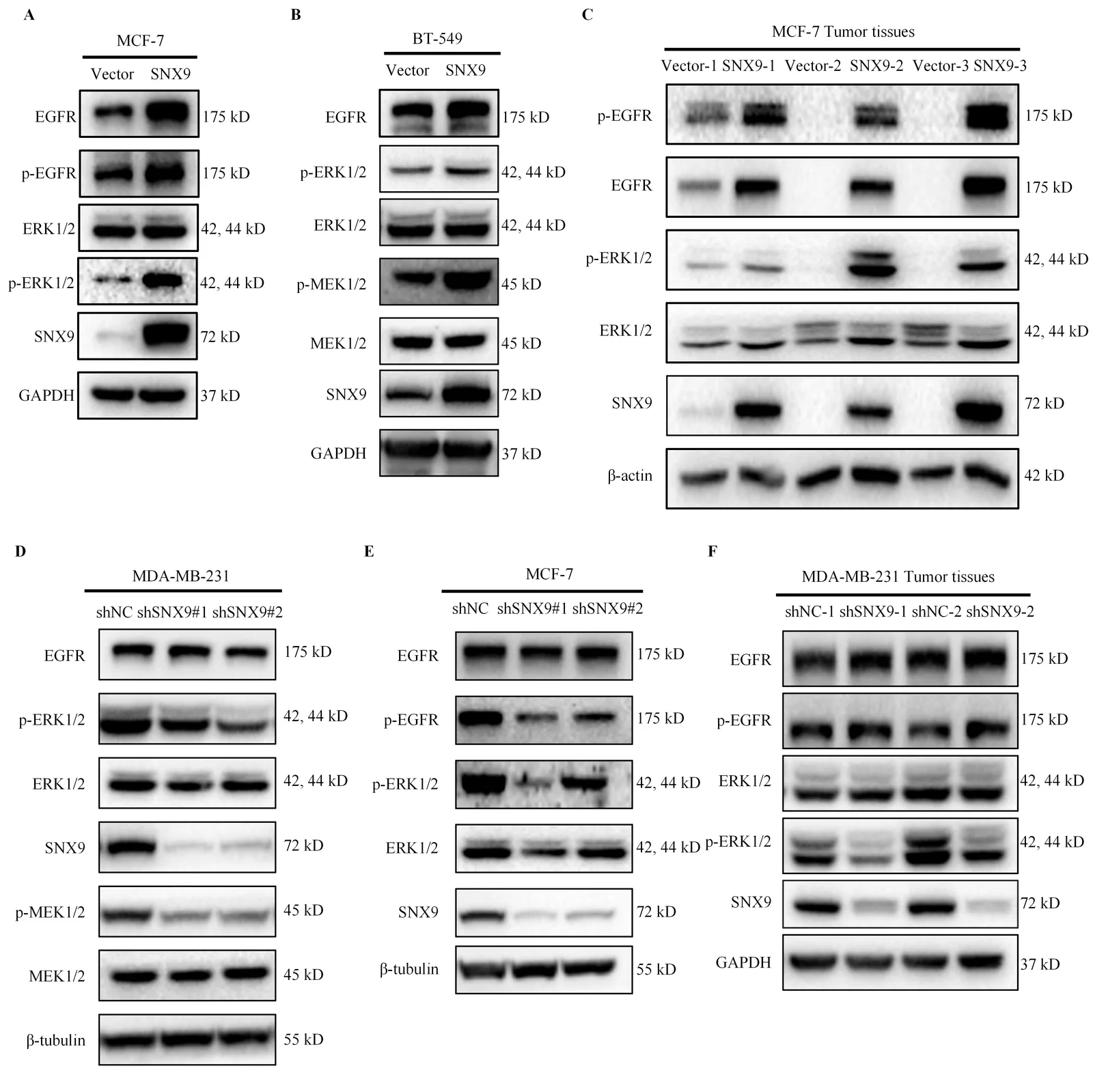
**SNX9 protein positively regulates the EGFR-ERK1/2 signaling module in mammary cancer.** (**A**–**C**) The expression of EGFR-ERK1/2 signaling pathway proteins in SNX9-OE (**A**) BT-549 and (**B**) MCF-7 cells, as well as in (**C**) SNX9-OE MCF-7 tumors, was tested by western blotting assay. (**D**–**F**) The corresponding analyses after SNX9 knockdown were performed in (**D**) MDA-MB-231 cells, (**E**) MCF-7 cells, and (**F**) MDA-MB-231 tumor samples. Abbreviations: SNX9, sorting nexin 9; EGFR, epidermal growth factor receptor; MEK1/2, mitogen-activated protein kinase kinase 1/2; ERK1/2, extracellular signal-regulated kinase 1/2; GAPDH, glyceraldehyde-3-phosphate dehydrogenase; P indicates phosphorylated form.

EGFR upregulation in BC is correlated with diverse oncogenic phenotypes, encompassing enhanced cell motility and proliferative outgrowth, as well as pro-angiogenic and metastatic activities [37,38,39]. Therefore, we hypothesized that the EGFR signalling pathway is responsible for the oncogenic properties of SNX9. Our findings demonstrate a positive regulatory function of SNX9 in the EGFR/ERK1/2 signalling cascade. Specifically, immunoblot analysis revealed that enforced SNX9 expression in MCF-7 (Fig. 6A) and BT-549 (Fig. 6B) cells substantially increased the phosphorylation of EGFR and ERK1/2. This promoting effect was further validated *in vivo*, as MCF-7 tumor tissues with SNX9 overexpression exhibited elevated p-EGFR and p-ERK1/2 levels versus the vector control group (Fig. 6C). Conversely, knockdown of SNX9 using shRNA (shSNX9#1 and shSNX9#2) in MDA-MB-231 and MCF-7 (Fig. 6D,E) led to a significant reduction of phosphorylated EGFR, Mitogen-activated protein kinase kinase 1/2 (MEK1/2), and ERK1/2. The repressive effect of SNX9 silencing on EGFR and ERK1/2 phosphorylation was also confirmed in MDA-MB-231 tumor tissues (Fig. 6F). Altogether, the above observations converge to show that SNX9 enhances the activation of the EGFR-mediated downstream signalling cascade (Supplementary Fig. S2).

**Figure 7:**
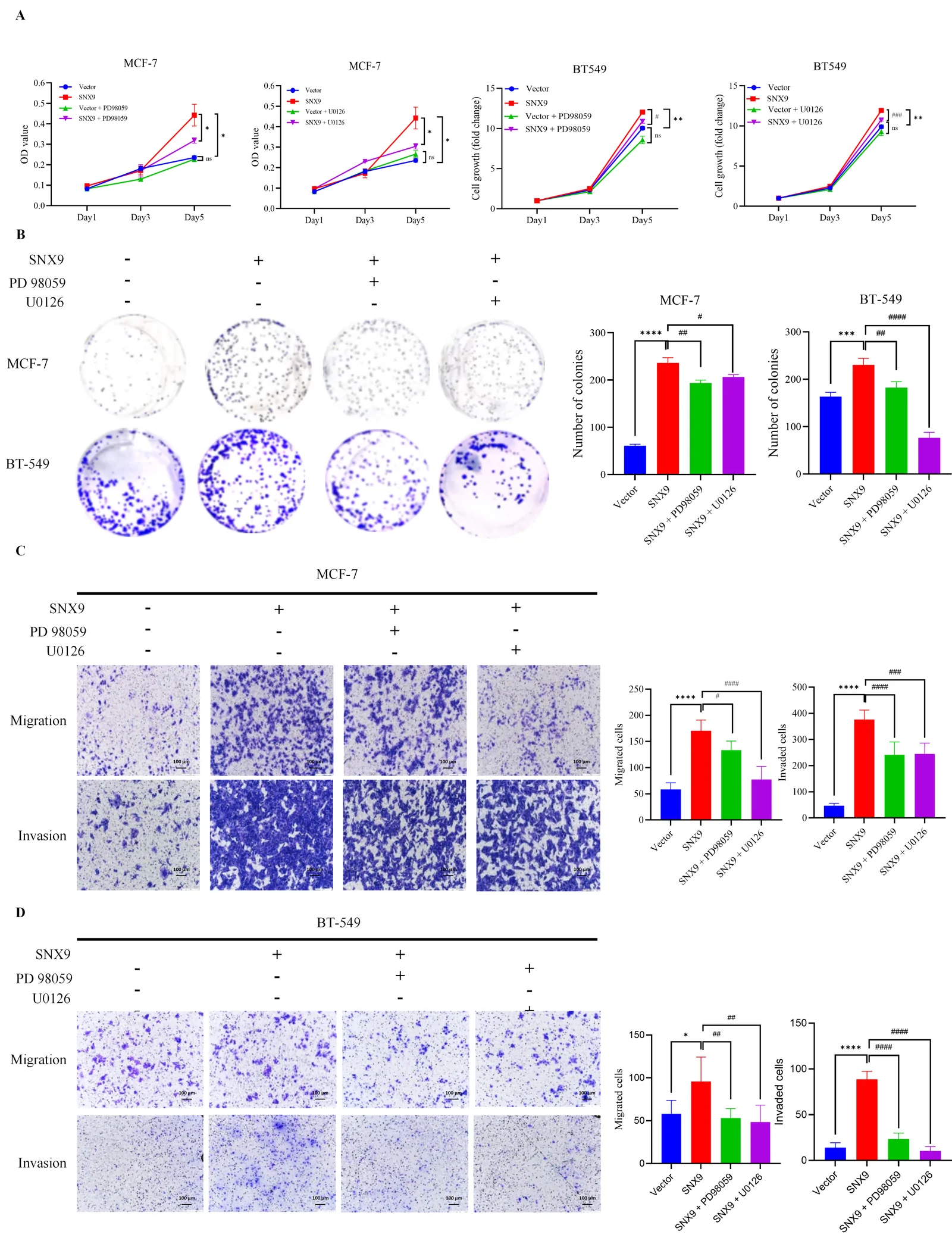
**SNX9 promotes breast cancer development via the EGFR-ERK1/2 signaling pathway.** SNX9-overexpressing MCF-7 and BT-549 cells were treated with MEK1/2 inhibitors (PD98059 at 25 μM or U0126 at 5 μM). (**A**) Cell growth and (**B**) colony-forming ability were assessed by the MTS and colony formation assays, respectively. (**C**) Cell migration and (**D**) invasion were assessed by the uncoated and Matrigel-coated transwell inserts, respectively (Scale bar: 100 μm). Differences between the two groups were examined by Student’s *t*-test, while differences among multiple groups were evaluated with one-way *ANOVA* (**A**–**D**). Values are mean ± SD. Asterisks denote comparisons between vector and SNX9-OE cells (*p < 0.05, **p < 0.01, ***p < 0.001, and ****p < 0.0001); hash symbols denote comparisons between untreated and inhibitor-treated OE groups (^#^*p* < 0.05; ^##^*p* < 0.01; ^###^*p* < 0.001; ^####^*p* < 0.0001); ns: not significant. Abbreviations: SNX9, sorting nexin 9.

We also examined whether blocking ERK1/2 activity could affect SNX9-mediated tumor promotion. SNX9-overexpressing cells were exposed to the MEK1/2 inhibitors (PD98059 and U0126) [40]. Both inhibitors attenuated the increases in proliferative potential and colony formation observed in SNX9-OE MCF-7 and BT-549 cells (Fig. 7A,B), and partially reduced their migratory and invasive capacities (Fig. 7C,D). These rescue experiment results indicate that the EGFR/ERK1/2 signalling pathway is, at least partially, responsible for SNX9’s function.

## 4 Discussion

This study revealed that SNX9 upregulation can enhance cancer cell invasiveness and metastatic potential both *in vitro* and *in vivo* by activating the EGFR-ERK1/2 signaling axis in BC cells, leading to increased proliferation and metastasis. SNX9 has a dual effect, being reduced in primary BC sites but increased in metastases [22,41], and survival analysis of TCGA data confirmed that higher SNX9 expression correlates with shorter survival time. This aligns with elevated SNX9 expression in metastatic sites, suggesting SNX9 functions as a tumor promoter rather than an initiator, particularly in metastasis. The strong correlation between SNX9 levels and metastatic progression suggests its utility in risk stratification; for example, SNX9 expression can be detected in tumor specimens to predict the risk of metastasis. In the present study, SNX9 depletion triggered G2/M cell cycle blockade, in agreement with a prior report [42] that SNX9 is involved in cytokinesis through endocytosis and chromosome alignment and segregation processes [42]. In this way, shSNX9 cells may stay in the mitosis process (metaphase), which increases population doubling time.

SNX9 was originally characterized as a modulator during the endocytic process mediated by clathrin [41]. Endocytosis has been demonstrated to be a crucial component of cell motility, resulting in actin remodeling, symmetrical cell elongation, and increased filopodia formation, ultimately promoting invasion and migration of transformed cells [41,43,44]. In this study, we found that SNX9 promotes BC cell invasion and migration by increasing cell motility, as evidenced by increased filopodia formation in SNX9-overexpressing cells and decreased pseudopodium formation in SNX9-silenced cells. Moreover, SNX9 was found to interact directly with TKS5, an invadopodia marker highly enriched in invasive protrusions (membrane structures) [45], further reinforcing its contribution in stimulating BC cell invasiveness and motility. *In vivo* models of lung metastasis and bone metastasis also revealed extended survival in the shSNX9 group compared to shNC.

In addition, the effects of SNX9 on cell movement and morphology were further confirmed by validating F-actin staining and active forms of RhoGTPases. MDA-MB-231 cells with downregulated SNX9 showed symmetrical spindle-shaped elongation but reduced locomotion ability. This elongation was similar to fibroblast-like cells, such as NIH-3T3 fibroblasts treated with Src tyrosine kinase knockout or c-Src inhibitors [46]. Since MDA-MB-231 cells share a similar morphology [23], it is possible that the Src/SNX9 axis is responsible for this phenomenon. Another explanation suggests that SNX9 interacts with phosphatidylinositol (PtdIns) lipids [41], and blockade of SNX9 impairs PI(3,4)P_2_-mediated endocytosis of integrin β3, leading to delayed focal adhesion turnover and reduced cell invasion events [24]. This may result in the persistence of integrins at focal adhesion, hindering the process of contraction at the trailing edge of cell movement. Thus, silencing SNX9 may prolong cell movement, although symmetrical elongation does not necessarily indicate a migratory phenotype. Migratory cells often have major protrusions at the leading edge, whereas elongated cells may exhibit uniform actin intensity [47]. The actin cytoskeleton and pseudopodia formation play a crucial role in dynamic cell motion events that respond to extracellular signals [48,49,50]. In *Drosophila* Schneider 2 cells, depletion of Sh3px1 caused defective lamellipodia formation [51]. Consistently, our work also found that SNX9-KD cells had a smaller lamellipodia area, while SNX9-OE BC cells had more membrane ruffles at the plasma membrane’s edge. SNX9 is a primary component of cellular filopodia, typically found on the tip and shaft, and regulates filopodia assembly [33,52].

The EGFR system is involved in normal cell growth and development [53], promoting proliferation, angiogenesis [54], invasion, metastasis [55], and inhibiting apoptosis [37,40]. In a loss-of-function screening model [56], several SNX proteins, including SNX9, were found to regulate EGFR degradative sorting [57]. Our data establish that SNX9 confers augmented proliferative, migratory, and invasive phenotypes in BC cells via EGFR-ERK1/2 signaling module activation. This study was in line with earlier studies that showed SNX5 overexpression enhances EGFR/ERK1/2 and protein kinase B (Akt) pathway activation in cancer cells [40,58,59]. In contrast, loss of SNX9 restrained BC cell growth and movement through attenuation of the EGFR-ERK1/2 cascade. SNX9 serves as a tumor-promoting factor, with increased expression leading to enhanced cell growth, migration, and invasion. Conversely, depletion of SNX9 results in altered cell morphology and reduced invasiveness. Additionally, SNX9 promotes pseudopodium formation through activation of Cdc42 and Rac1. The EGFR-ERK1/2 signaling pathway is also implicated in SNX9-induced cell proliferation and motility, as demonstrated by the partial reversal of these effects with MEK1/2 inhibitors (Fig. 7). The findings imply that SNX9 may act as a context-dependent molecular coordinator of BC progression rather than as a purely linear signaling factor. Additional time-course and pathway-dissection studies will be needed to determine how these programs are temporally and mechanistically coordinated. Thus, SNX9 could serve as a viable therapeutic target for BC intervention.

While this study elucidates the function of SNX9 in BC development through EGFR/ERK signaling and actin cytoskeleton remodeling, several limitations should be acknowledged. Firstly, our bioinformatics and primary tumor data suggest that SNX9 upregulation correlates with poor prognosis, but validation in metastatic lesions (e.g., via IHC of patient-derived metastases or circulating tumor cells) was beyond the scope of this project. Future studies should directly compare SNX9 levels in primary tumors versus matched metastases to clarify its stage-specific roles. Secondly, although statistically significant differences were observed, the *in vivo* findings were derived from a limited cohort. Future studies should employ larger sample sizes to reinforce the robustness of these results before clinical extrapolation. Also, while our study focused primarily on EGFR/ERK signaling, the potential involvement of parallel pathways, such as WNT/β-catenin, cannot be ruled out. Future studies employing multiplexed kinase assays (e.g., kinase chip experiments) together with integrative omics approaches will be essential to dissect the broader signaling network orchestrated by SNX9. Last but not least, we demonstrate that SNX9 activates the EGFR/ERK1/2 pathway, but the specific trafficking mechanism (whether SNX9 promotes EGFR signaling by enhancing receptor recycling to the cell surface or primarily by preventing degradative sorting of EGFR into lysosomes) remains unclear. Mechanistically, prior studies have shown that SNX9 interacts with AP-2, clathrin, and dynamin-2, and localizes predominantly to clathrin-coated structures at the plasma membrane, rather than to EEA1-positive early endosomes, supporting its role in the early stages of clathrin-mediated endocytosis [20,60]. On the other hand, previous EGFR trafficking studies have shown that clathrin-associated internalization can preserve a signaling-competent receptor pool and favor sustained signaling/recycling [61], whereas impaired progression from early to late endosomes delays lysosomal degradation and prolongs EGFR signaling [62]. Taken together, our data suggest that SNX9 may enhance EGFR/ERK signaling by sustaining a clathrin-associated signaling-competent pool of EGFR. Nevertheless, whether this effect is primarily attributable to enhanced receptor recycling or delayed delivery to degradative late endosome/lysosome compartments cannot be determined from the current dataset.

Future translational directions could involve exploring small molecule inhibitors or RNA-based therapeutics targeting SNX9. Existing agents that target endocytosis or actin remodeling [63] might indirectly reduce SNX9-driven metastasis. Additionally, since SNX9 may regulate intracellular transport of chemotherapeutic drugs, its inhibition could resensitize resistant tumors to therapies [64,65]. An additional translational implication of our findings emerges from the CAPmed-BC (Cold Atmospheric Plasma Medicine for Breast Cancer) database. Analysis of the CAPmed-BC database shows that SNX9 is significantly altered in TNBC cells at 8 h post-CAP exposure, but not at 1 h or in non-TNBC models. Given that CAP sensitivity [66,67] in TNBC involves EGFR signaling—a pathway potentiated by SNX9—our findings suggest a potential role for SNX9 in CAP responsiveness. However, direct evaluation in CAP-treated models is required to confirm SNX9 as a mediator or biomarker.

## 5 Conclusion

**Figure 8:**
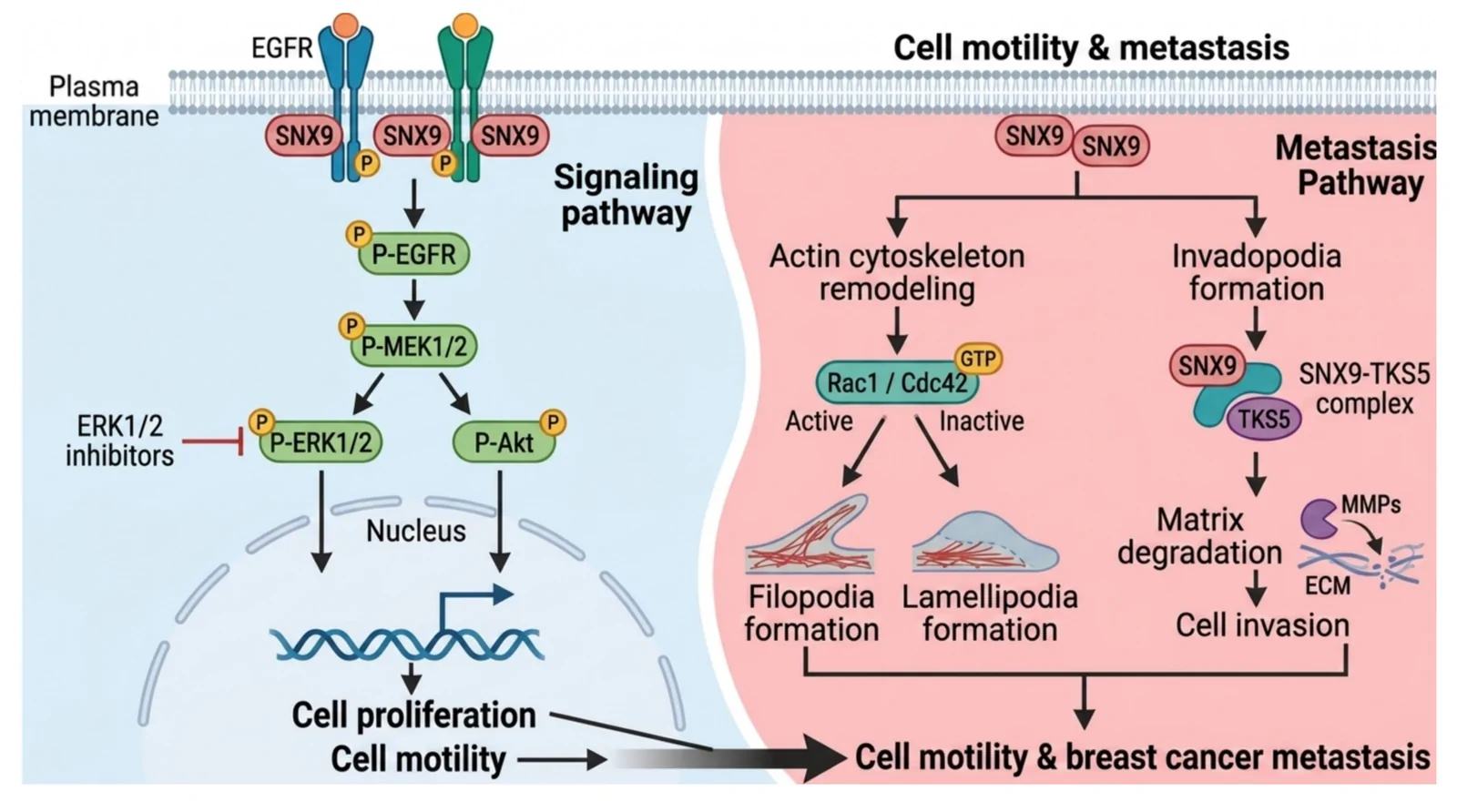
**SNX9’s mechanism in regulating cell proliferation and motility in breast cancer.** SNX9 overexpression promoted BC cell proliferation, migration, and invasion, whereas its silencing exerted reciprocal suppressive effects. Active small Rho-GTPases and the EGFR-ERK1/2 signaling pathway were involved in the mechanisms of SNX9-induced cell proliferation and motility. Abbreviations: SNX9, sorting nexin 9; EGFR, epidermal growth factor receptor; MEK1/2, mitogen-activated protein kinase kinase 1/2; ERK1/2, extracellular signal-regulated kinase 1/2; AKT, protein kinase B; Rac1, Ras-related C3 botulinum toxin substrate 1; Cdc42, cell division control protein 42 homolog; GTP, guanosine triphosphate; TKS5, tyrosine kinase substrate with five SH3 domains; MMPs, matrix metalloproteinases; ECM, extracellular matrix. P indicates phosphorylated form.

This work positions SNX9 as an essential regulator of BC progression, with its pro-tumorigenic effects mediated by activating the EGFR/ERK1/2 signaling cascade to enhance tumor cell proliferation and metastasis, while simultaneously promoting actin cytoskeleton remodelling through interactions with TKS5 and modulation of RhoGTPases (Cdc42/Rac1) to facilitate invasive pseudopodia formation (Fig. 8). Clinically, SNX9 overexpression is strongly associated with poor patient survival, positioning it as a prognostic factor and a therapeutic candidate. Together, these observations identify a dual regulatory mechanism by which SNX9 coordinates signalling and cytoskeletal dynamics to fuel aggressive breast cancer phenotypes, providing a foundational framework for future investigations into SNX9-targeted therapies, particularly in metastatic settings where current treatment options remain limited.

## Data Availability

The data are available from the corresponding authors upon reasonable request.

## Supplementary Materials

The supplementary material is available online at https://www.techscience.com/doi/10.32604/or.2026.082536/s1.

## List of Abbreviations

ADAM9	A Disintegrin and Metalloprotease 9
ANOVA	Analysis of Variance
ATCC	American Type Culture Collection
Akt	protein kinase B
BC	Breast cancer
BrdU	Bromodeoxyuridine
CDK2	Cyclin-dependent kinase 2
CDK6	Cyclin-dependent kinase 6
CDKI	Cyclin-dependent kinase inhibitor
Cdc42	Cell Division Cycle 42 homolog
E2F	the adenoviral early region 2 binding transcription factor
ECM	Extracellular matrix
EGFR	Epidermal growth factor receptor
ER	Estrogen Receptor
ERK1/2	Extracellular signal-regulated kinase 1/2
FBS	Fetal Bovine Serum
GAPDH	Glyceraldehyde-3-Phosphate Dehydrogenase
H&E	Hematoxylin and Eosin
HER2	Human Epidermal Growth Factor Receptor 2
IF	Immunofluorescence
IHC	Immunohistochemistry
IP	Immunoprecipitation
KD	Knockdown
MEK1/2	Mitogen-activated protein kinase kinase 1/2
MMPs	Matrix Metalloproteinases
OD	Optical Density
OE	Overexpression
PAK-PBD	p21 Activated kinase-p21 Binding Domain
PCNA	Proliferating Cell Nuclear Antigen
PFA	Paraformaldehyde
PgR	Progesterone Receptor
PI	Propidium iodide
P/S	Penicillin-Streptomycin
PtdIns	Phosphatidylinositol
PX	Phox homology
RT-qPCR	Reverse Transcription Quantitative Polymerase Chain Reaction
Rac1	Ras-related C3 botulinum toxin substrate 1
RhoGTPase	Rho guanosine triphosphatase
RNA	Ribonucleic acid
RB	Retinoblastoma
SD	Standard Deviation
SNX9	Sorting nexin 9
shRNAs	short hairpin RNAs
TNBC	Triple-negative breast cancer
TKS5	Tyrosine kinase substrate with five SH3 domains
