# Supplementary material for: SNX9 Orchestrates Lung Metastasis via EGFR-ERK Signaling and Actin Cytoskeleton Remodeling in Breast Cancer
Source: Oncol Res. 2026 Sep 14;34(10):22. doi: 10.32604/or.2026.082536 (PMC13591323; doi:10.32604/or.2026.082536)
Supplement: Supplementary file 1 [file OncolRes-34-82536-s001.zip › TSP_OR_82536-s001.docx]

**Supplementary Figure S1. Quantitative analysis of cell motility and invasion in breast cancer cells with SNX9 overexpression or knockdown. Related to Figure 4. (A-F)** The 2D migration ability of **(A)** BT-549 SNX9-OE cells and **(D)** MDA-MB-231 SNX9-KD cells were measured by wound healing assay. The effect of **(B)** SNX9-OE cells and **(E)** SNX9-KD cells on cell 3D migration ability was assessed by transwell assay, without coating Matrigel. The effect of **(C)** SNX9 OE cells and **(F)** SNX9-KD cells on cell invasion ability was assessed by Matrigel-coated transwell assay. Data were analyzed by Student’s *t*-test (**A, B, C**) for comparison between two groups and one-way *ANOVA* (**D, E, F**) for comparisons involving more than two groups. Data were presented as mean ± SD. **p* <0.05, ***p*＜0.01, ****p*＜0.001, and *****p* < 0.0001 versus to the control group.

**Supplementary Figure S2. Quantitative analysis of EGFR/ERK signaling pathway markers with SNX9 overexpression or knockdown in breast cancer cells and tumor tissues. Related to Figure 6.** The expression level of EGFR/ERK signaling pathway markers and SNX9 **(A)** in SNX9-OE MCF-7 cells, **(B)** SNX9-OE BT-549 cells, **(C)** in SNX9-OE MCF-7 tumor tissues, **(D)** in SNX9-KD MDA-MB-231 cells, **(E)** SNX9-KD MCF-7 cells and **(F)** SNX9-KD MDA-MB-231 tumor tissues were measured by Western blot assay. Data were analyzed by Student’s *t*-test (**A, B, C, F**) for comparison between two groups and one-way *ANOVA* (**D, E**) for comparisons involving more than two groups. Data are mean ± SD. **p* <0.05, ***p*＜0.01, ****p*＜0.001, and *****p* < 0.0001 versus to the control group. ns: not significant.
